# Effects of Erythropoietin Administration on Adrenal Glands of Landrace/Large White Pigs after Ventricular Fibrillation

**DOI:** 10.1155/2016/7261960

**Published:** 2016-07-18

**Authors:** Armando Faa, Gavino Faa, Apostolos Papalois, Eleonora Obinu, Giorgia Locci, Maria Elena Pais, Pavlos Lelovas, Dimitrios Barouxis, Charalampos Pantazopoulos, Panagiotis V. Vasileiou, Nicoletta Iacovidou, Theodoros Xanthos

**Affiliations:** ^1^Department of Surgery, Section of Pathology, University of Cagliari, Cagliari, 09100 Sardinia, Italy; ^2^ELPEN Experimental Center, Athens, Greece; ^3^Medical School, National and Kapodistrian University of Athens, Athens, Greece; ^4^Neonatal Department, Aretaieio Hospital, Medical School, National and Kapodistrian University of Athens, Athens, Greece; ^5^European University Cyprus, Nicosia, Cyprus

## Abstract

*Aim.* To evaluate the effects of erythropoietin administration on the adrenal glands in a swine model of ventricular fibrillation and resuscitation.* Methods*. Ventricular fibrillation was induced* via* pacing wire forwarded into the right ventricle in 20 female Landrace/Large White pigs, allocated into 2 groups: experimental group treated with bolus dose of erythropoietin (EPO) and control group which received normal saline. Cardiopulmonary resuscitation (CPR) was performed immediately after drug administration* as per* the 2010 European Resuscitation Council (ERC) guidelines for Advanced Life Support (ALS) until return of spontaneous circulation (ROSC) or death. Animals who achieved ROSC were monitored, mechanically ventilated, extubated, observed, and euthanized. At necroscopy, adrenal glands samples were formalin-fixed, paraffin-embedded, and routinely processed. Sections were stained with hematoxylin-eosin.* Results.* Oedema and apoptosis were the most frequent histological changes and were detected in all animals in the adrenal cortex and in the medulla. Mild and focal endothelial lesions were also detected. A marked interindividual variability in the degree of the intensity of apoptosis and oedema at cortical and medullary level was observed within groups. Comparing the two groups, higher levels of pathological changes were detected in the control group. No significant difference between the two groups was observed regarding the endothelial changes.* Conclusions*. In animals exposed to ventricular fibrillation, EPO treatment has protective effects on the adrenal gland.

## 1. Introduction

Cardiac arrest is one of the leading causes of death. After return of spontaneous circulation (ROSC), post-cardiac arrest syndrome represents the predominant disorder in survivors. Besides the postarrest brain injury, the postresuscitation myocardial stunning, and the systemic ischemia/reperfusion response, this syndrome is characterized by adrenal insufficiency, a disorder that often lacks attention and appears to be associated with a poor prognosis in cases of postresuscitation shock [1].

The hormone erythropoietin (EPO), from Greek: $\varepsilon \rho \upsilon \theta \rho \overset{´}{o}\varsigma$ (red) and $\pi ο\iota \varepsilon \tilde{\iota }\nu$ (make), controls red blood cell production in the human body. It is a cytokine crucial for erythrocyte precursors production in the fetal liver and, after birth, in the bone marrow. Human EPO has a molecular weight of 34 kDa. The hematopoietic cytokine EPO is produced by the kidney in response to hypoxia and stimulates erythroid progenitor cells to increase the number of mature red blood cells, thereby increasing the O_2_ carrying capacity.

It is well established that EPO-receptors are widespread in cells throughout the body, including endothelial cells, myocardiocytes, macrophages, retinal cells, and cells of the adrenal cortex [2].

Erythropoietin treatment influences directly or indirectly the function of endocrine organs; it induces a significant decrease in somatotropin, prolactin, follitropin, lutropin, ACTH, cortisol, plasma renin activity, aldosterone, noradrenaline, adrenaline, dopamine, glucagon, pancreatic polypeptide, and gastrin plasma levels. It also increases plasma insulin, estradiol, testosterone, atrial natriuretic peptide, thyrotropin, and thyroxine levels [3].

Recent studies suggest that adrenal insufficiency might be due to adrenal microvessel contraction; adrenomedullin, a vasodilator peptide with a half-life of about 20 min, might partly be responsible for this phenomenon as it dampens baroreflex-driven responses and buffers sympathetic actions.

As it has been hypothesized that EPO might have a multiorgan protection role [4], the present study aimed at better understanding of the pathophysiology of adrenal insufficiency and the possible protective effects of EPO at adrenal level, in an experimental animal model of ventricular fibrillation (VF).

## 2. Materials and Methods

The protocol was approved by the Directorate of Veterinary Services of Prefecture of Athens, Attica, Greece, according to Greek legislation regarding ethical and experimental procedures. Twenty female Landrace/Large White pigs, aged 10–15 weeks with an average weight of 19 ± 2 kg, all from the same breeder (Validakis, Athens, Greece) were the study subjects. All animals were prepared in a standardized fashion at ELPEN Experimental-Research Center, Pikermi, Greece, as previously described [5]. Initial sedation was achieved by intramuscular injection of ketamine hydrochloride (10 mg/kg), midazolam (0.5 mg/kg), and atropine (0.05 mg/kg). Anaesthesia was induced with an intravenous (iv) bolus dose of propofol (2 mg/kg)* via* the marginal auricular vein. The pigs were then intubated and mechanically ventilated with a volume-controlled ventilator. End-tidal CO_2_ (ETCO_2_) was monitored by waveform capnography, and respiratory frequency was adjusted to maintain ETCO_2_ between 35 and 40 mm Hg. A bolus dose of cis-atracurium (0.15 mg/kg) was administered to ascertain synchrony with the ventilator. Continuous infusion of propofol 150 *μ*g/kg/min was used to maintain adequate anaesthetic depth and fentanyl 4 *μ*g/kg to ensure analgesia. Cardiac rhythm and heart rate were monitored by electrocardiography. Right carotid artery and right internal jugular vein were catheterized, and aortic pressure was measured using a fluid-filled catheter. Mean arterial pressure (MAP) was determined by electronic integration of the aortic blood pressure waveform. A catheter was inserted into the right atrium* via* the right jugular vein for continuous measurement of right atrial pressure.

### 2.1. Experimental Protocol

After surgery, the animals were allowed a 30-minute stabilization period, before baseline data were collected. Ventricular fibrillation was induced with a 9 V ordinary cadmium battery* via* a pacing wire forwarded into the right ventricle through the cannulated right jugular vein, as previously described [6, 7], and was confirmed by electrocardiography and by a sudden drop in MAP. Mechanical ventilation and administration of anaesthetics were discontinued simultaneously with the onset of VF and the animals were left untreated for 8 min. A bolus dose of adrenaline (0.02 mg/kg) was then administered, and the animals (*n* = 10 per group) were randomly treated either with a bolus dose of EPO or with normal saline as placebo. All drugs were injected* via* the marginal auricular vein, followed by a 10 mL normal saline flush to assist faster circulation of medications. The researchers were blinded to the animal's allocation, until the experiment was completed and all haemodynamic and survival data were collected. Cardiopulmonary resuscitation was commenced immediately after drug administration. Mechanical ventilation was resumed with 21% oxygen and automatic continuous precordial compression was initiated at a rate of 100 per minute following the two-minute cycles* as per* the 2010 ERC ALS guidelines [5]. After 2 min of CPR, defibrillation was attempted with a 4 J/kg monophasic shock. Cardiopulmonary resuscitation was resumed for another 2 minutes after each defibrillation attempt. Further bolus doses of adrenaline (0.02 mg/kg) were administered every 4 minutes during CPR. Each experiment continued until ROSC or if asystole/pulseless electrical activity (PEA) occurred >10 min after CPR initiation. Return of spontaneous circulation was defined as the presence of a perfusing cardiac rhythm with a mean arterial pressure of at least 60 mmHg for a minimum of 5 minutes. After ROSC, the animals were monitored closely and mechanically ventilated for 6 hours, under general anaesthesia, with the prearrest settings. No other interventions were made after ROSC. After 6 hours, all catheters were removed; the animals were allowed to recover from anaesthesia and were extubated and transferred to their observation cages. They remained under observation for 48 hours after ROSC before euthanasia with an iv bolus dose of propofol 40 mg, followed by 2 gr thiopental iv. Experimental endpoints were ROSC and 48 h survival.

Histological analyses were carried out in all treated animals. Adrenal tissue samples were fixed in 10% formalin, routinely processed, and paraffin-embedded; the initial block was cut into 6-7 blocks about 2-3 mm wide. Five-micron-thick sections were deparaffinized and hydrated to water. They were then colored with hematoxylin for 15 minutes, washed in running tap water for 20 minutes, and counterstained with eosin for 15 seconds to 2 minutes. Finally, slides were dehydrated in 95% absolute alcohol and cleared in xylene. All hematoxylin-eosin- (H&E-) stained slices were assessed by a pathologist who was blinded to the animal treatment and outcome. For the quantification of the elementary lesions observed in adrenal glands, five fields were randomly selected and observed for the count in each sample. The variables are expressed as mean ± standard deviation (SD) for each elementary lesion found at histology. Repeated Student's *t*-tests were used to evaluate any possible significant statistical difference between the control group and the experimental group.

## 3. Results

The histological study of adrenal glands of the pigs submitted to VF revealed pathological changes in all animals (see Table 1). The most important changes were oedema and apoptosis of adrenal cells. Moreover, morphological signs of mild and focal endothelial cell damage were detected. All pathological changes were detected both in the adrenal cortex and in the medulla. A marked interindividual variability regarding the degree of adrenal pathology was observed among the animals within the same group: in particular, significant differences were observed among animals of the same group regarding the intensity of apoptosis and oedema at cortical and medullary level. No significant interindividual variability was detected regarding the degree of endothelial changes (see Table 1).

**Figure 1:**
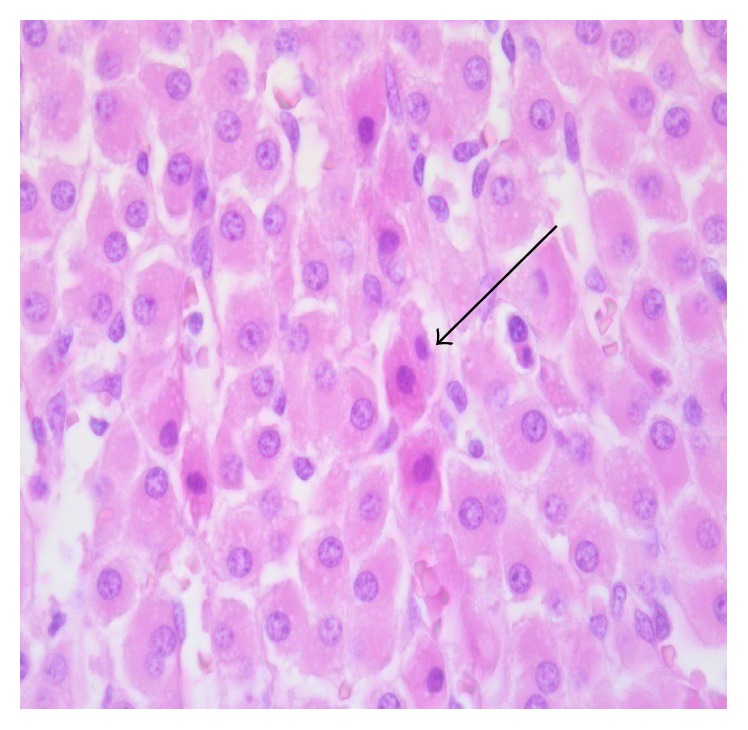
Diffuse apoptosis in the cortex of the adrenal glands. Cells undergoing apoptosis are characterized by cell detachment, chromatin condensation, and eosinophilia of the cytoplasm.

**Figure 2:**
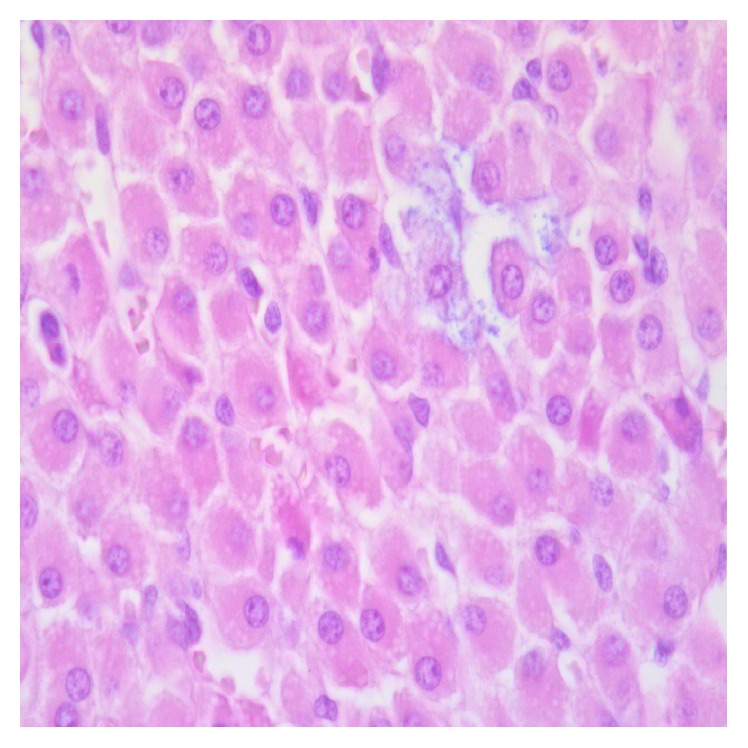
Diffuse oedema in the adrenal cortex. Due to the increase of the interstitial fluid, adrenal cells are detached from each other.

**Figure 3:**
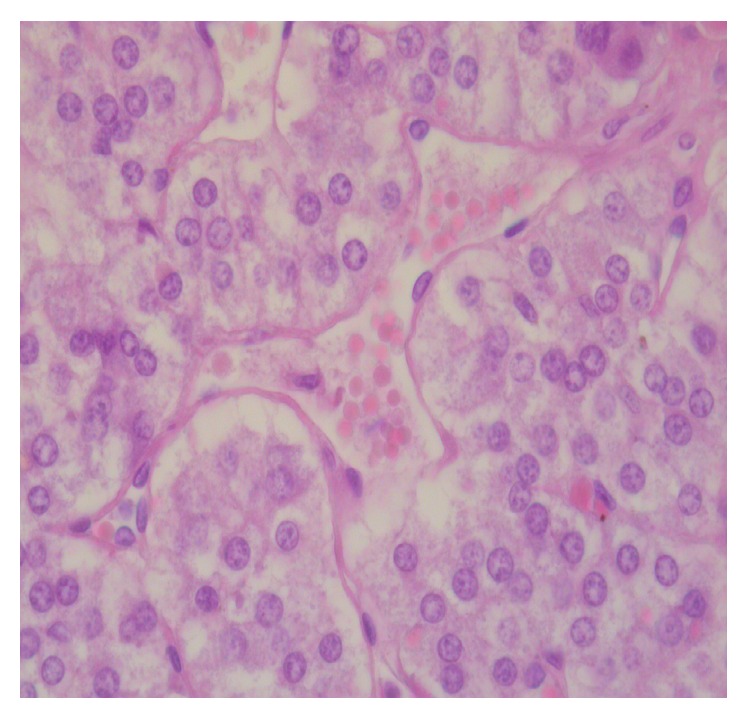
Mild endothelial damage in adrenal vessel, represented by swelling of the nuclei of endothelial cells (center of the picture).

Differences regarding the intensity and diffusion of the pathological changes between the two groups of animals were detected. The adrenal glands of the animals of the control group (group 1) were characterized by higher levels of pathological changes. In particular, apoptosis of cortical adrenal cells was present in the vast majority of animals in this group (Figure 1), contrarily with the presence of scattered apoptotic cells in animals submitted to EPO treatment (group 2). Differences were also detected regarding oedema, which was diffuse in all animals of group 1 (Figure 2), whereas in the EPO-treated animals cortical oedema was focal or mild in the majority of animals (see Table 1). No significant differences between the two groups were found regarding the endothelial changes (mainly endothelial swelling), which were mild (Figure 3) and focally detected in similar degree in the cortex of animals of groups 1 and 2 (see Table 1).

**Figure 4:**
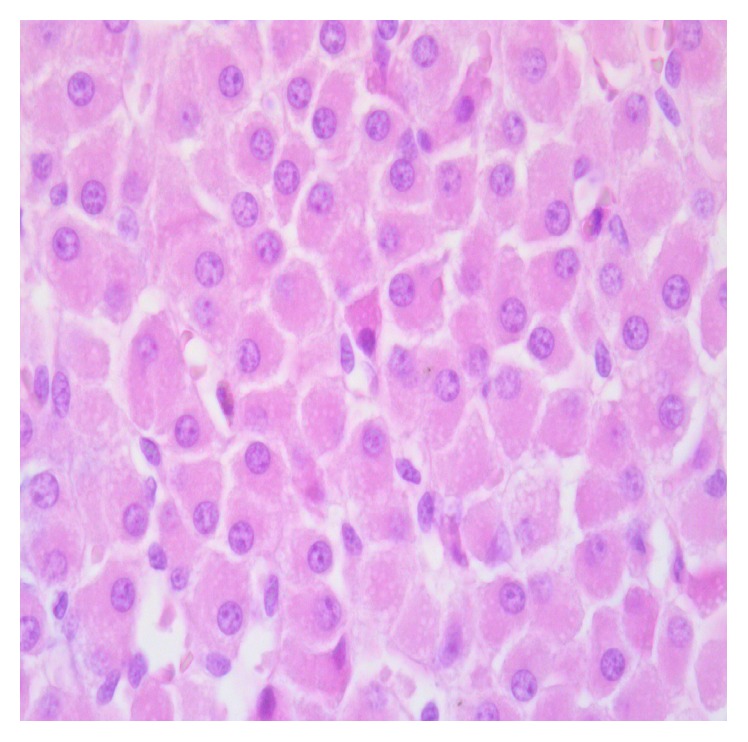
Apoptosis of the adrenal medulla. The scattered cells undergoing apoptosis show a picknotic nucleus and a dense eosinophilic cytoplasm.

Histological examination of the adrenal medulla showed minor pathological changes, compared with the adrenal cortex. Apoptosis was focal, appearing as scattered apoptotic cells showing a hypereosinophilic cytoplasm and a dark nucleus. Cells undergoing apoptosis appeared intermingled with normal-appearing larger medullary cells (Figure 4). No significant difference regarding the degree of medullary apoptosis was detected between the two groups (see Table 1). Oedema was found in the adrenal medulla of all the animals, including those treated with EPO (Table 1).

Regarding the endothelial lesions detected in the medulla, endothelial swelling was the unique lesion focally detected in both groups.

To minimize errors due to sampling variability, the count of histological changes was based on the observation of five high power-fields, randomly selected from adrenal cortex and the medulla of the collected adrenal samples.

## 4. Discussion

In a previous preliminary study from our team, EPO administration resulted in higher rates of ROSC and higher 48 h survival with improved haemodynamics throughout the resuscitation period [8]. In other studies of experimental model of cardiac ischemia-reperfusion injury, EPO administration had contradictory effects: on one hand, EPO treatment increased functional recovery, suggesting the need of additional studies to evaluate therapeutic applications of EPO administration [4]; on the other hand, EPO did not have any significant protecting effect on cardiomyocytes when exposed to VF [8]. Moreover, EPO was demonstrated to prevent apoptosis of motor neurons in an experimental model of spinal cord ischemic injury [9]. The antiapoptotic role of EPO was also reported in a rat model of cerebral ischemia, where systemic administration of EPO after middle-cerebral artery occlusion dramatically reduced the volume of brain infarction and promoted cell survival by protecting neurons against hypoxia-induced cell death [10]. Conflicting results have recently been reported regarding the mechanisms of neuroprotection of EPO treatment. In particular, the neuroprotective effects of EPO are not restricted to its antiapoptotic role, given the lack of Akt activation after EPO administration. An alternative mechanism, proposed to explain EPO neuroprotection, was blood-brain barrier preservation and prevention of brain oedema [11].

The protective effects of EPO treatment are not restricted to the central nervous system. Pretreatment with EPO has recently been shown to ameliorate the renal injury caused by bilateral renal ischemia-reperfusion, by attenuating oxidative stress in kidney cells [12]. Moreover, the protective effect of EPO on kidney cells was increased by its association with melatonin in a rat model of ischemia-reperfusion injury [13].

With the present study we demonstrate, for the first time to the best of our knowledge, that EPO treatment has protective effects on the adrenal gland injury following VF. This protection was mainly observed in the adrenal cortex. It was evidenced in the medulla but in a minor degree. In the pigs of the control group, oedema was intense and diffuse in the cortex of all animals, indicating the increase in interstitial fluid as a possible mechanism for adrenal gland pathology. In contrast, in animals treated with EPO, oedema of the adrenal cortex was focal and mild in the majority of animals, reaching levels similar to those detected in all animals of the other group only in 2 out of 10 animals.

Striking differences were also found regarding apoptosis of cortical adrenal cells. High levels (degree 3 or 4) of cell shrinkage and cell detachment, typical morphological signs of apoptotic cell death, were observed in 7 out 9 animals of the first group, not receiving EPO treatment. Contrarily, similar high levels of apoptosis were observed in 2 out of 10 animals in the EPO-treated animals (group 2). These data clearly indicate that EPO protects adrenal cortical cells by limiting apoptosis and interstitial oedema. The origin of oedema is not clear, on the basis of our findings. No significant difference between the 2 groups was found, in this study, regarding the occurrence of vascular damage and, in particular, of endothelial damage. Endothelial changes (focal endothelial swelling) were mild in all the animals. As for the levels of endothelial damage, no difference was found between groups, both in the cortical and in the medullary zones.

In our study, the protective effects of EPO treatment were mainly observed in the cortical zones. Apoptotic cells were observed to be scattered even in the medulla, suggesting the existence of mild medullary pathological changes due to VF. The absence of any difference, regarding apoptosis of medullary cells, between the 2 groups might suggest that the antiapoptotic protective effects of EPO in this experimental model are restricted to cortical adrenal cells. These findings might be related to previous studies on an uneven distribution of EPO-receptors in the adrenal glands, indicating a preferential localization of EPO-receptors in the adrenal cortex [3].

Some differences were observed between the 2 groups regarding the degree of oedema in the medulla: the finding of a mild and focal oedema (grade 1) in the medulla of 7 out of 10 animals in the EPO-treated group contrasted with the detection of grade 1 oedema in 2 out of 9 animals of the control group. These findings confirm previous studies, suggesting that the protective role of EPO should not be restricted to its antiapoptotic effect [9], and lay stress on the ability of EPO treatment to end or decrease the levels of interstitial oedema in the adrenal gland, as previously reported in the brain [11].

There are significant limitations in the current study. This is an animal study and extrapolation to humans should be done with caution. Moreover, the number of animals was relatively low and in the current study we did not measure cytokines and stress hormones' levels such as epinephrine and norepinephrine.

In conclusion, our study clearly shows that EPO treatment during resuscitation of Landrace/Large White pigs submitted to VF has a clear protective effect on adrenal glands. The mechanism of this action might be multifactorial, resulting in a marked decrease in the degree of apoptosis of cortical adrenal cells and a decrease in oedema, both in the cortex and in the adrenal medulla.

## Figures and Tables

**Table 1:** 

Cases	Cortical			Medulla		
	Apoptosis	Oedema	Endothelial damage	Apoptosis	Oedema	Endothelial damage
1	2	3	1	1	2	1
2	2	3	1	1	1	1
4	3	3	1	1	1	1
5	3	3	1	1	2	1
6	4	3	1	1	2	1
7	4	3	1	1	2	1
8	4	3	1	1	2	1
9	3	3	1	1	2	1
10	4	3	1	1	2	1
12	2	2	1	1	1	1
13	3	2	1	1	1	1
14	3	3	1	1	1	1
15	2	3	1	1	2	1
15B	2	2	1	1	2	1
15C	2	2	1	1	1	1
16	2	1	1	1	1	1
17	1	1	1	1	1	1
18	1	2	1	1	1	1
20	1	2	1	1	2	1

## References

[1] Pene F., Hyvernat H., Mallet V. (2005). Prognostic value of relative adrenal insufficiency after out-of-hospital cardiac arrest. *Intensive Care Medicine*.

[2] Juul S. E., Yachnis A. T., Christensen R. D. (1998). Tissue distribution of erythropoietin and erythropoietin receptor in the developing human fetus. *Early Human Development*.

[3] Kokot F., Wiecek A., Schmidt-Gayk H. (1995). Function of endocrine organs in hemodialyzed patients of long-term erythropoietin therapy. *Artificial Organs*.

[4] Cai Z., Manalo D. J., Wei G. (2003). Hearts from rodents exposed to intermittent hypoxia or erythropoietin are protected against ischemia-reperfusion injury. *Circulation*.

[5] Xanthos T., Lelovas P., Vlachos I. (2007). Cardiopulmonary arrest and resuscitation in Landrace/Large White swine: a research model. *Laboratory Animals*.

[6] Rao S. V., Stamler J. S. (2002). Eythropoietin , anemia, and orthostatic hypotension: the evidence mounts. *Clinical Autonomic Research*.

[7] Parsa C. J., Kim J., Riel R. U. (2004). Cardioprotective effects of erythropoietin in the reperfused ischemic heart: a potential role for cardiac fibroblasts. *The Journal of Biological Chemistry*.

[8] Vasileiou P. V. S., Xanthos T., Barouxis D. (2014). Erythropoietin administration facilitates return of spontaneous circulation and improves survival in a pig model of cardiac arrest. *American Journal of Emergency Medicine*.

[9] Celik M., Gökmen N., Erbayraktar S. (2002). Erythropoietin prevents motor neuron apoptosis and neurologic disability in experimental spinal cord ischemic injury. *Proceedings of the National Academy of Sciences of the United States of America*.

[10] Sirén A.-L., Fratelli M., Brines M. (2001). Erythropoietin prevents neuronal apoptosis after cerebral ischemia and metabolic stress. *Proceedings of the National Academy of Sciences of the United States of America*.

[11] Ratilal B. O., Arroja M. M. C., Rocha J. P. F. (2014). Neuroprotective effects of erythropoietin pretreatment in a rodent model of transient middle cerebral artery occlusion. *Journal of Neurosurgery*.

[12] Elshiekh M., Kadkhodaee M., Seifi B., Ranjbaran M., Ahghari P. (2015). Ameliorative effect of recombinant human erythropoietin and ischemic preconditioning on renal ischemia reperfusion injury in rats. *Nephro-Urology Monthly*.

[13] Ahmadiasl N., Banaei S., Alihemati A., Baradaran B., Azimian E. (2014). Effect of a combined treatment with erythropoietin and melatonin on renal ischemia reperfusion injury in male rats. *Clinical and Experimental Nephrology*.

